# Rethinking Ecosystem Resilience in the Face of Climate Change

**DOI:** 10.1371/journal.pbio.1000438

**Published:** 2010-07-27

**Authors:** Isabelle M. Côté, Emily S. Darling

**Affiliations:** Department of Biological Sciences, Simon Fraser University, Burnaby, British Columbia, Canada

Resilience is usually defined as the capacity of an ecosystem to absorb disturbance without shifting to an alternative state and losing function and services [1]–[3]. The concept therefore encompasses two separate processes: resistance—the magnitude of disturbance that causes a change in structure—and recovery—the speed of return to the original structure [4],[5]—which are fundamentally different but rarely distinguished. Yet, resilience has become a central concept in the management of natural ecosystems [6],[7]. Many current management actions aim to alleviate local stressors in an effort to increase ecosystem resilience to global climate change [8],[9]. Such a management philosophy is premised on the belief that eliminating local drivers of ecological change will increase the ability of an ecosystem to resist future climate disturbances, its ability to recover from such disturbances, or both [2],[6]. Measuring resilience is fraught with difficulties [1],[3]. Nevertheless, assessing changes in resilience as a result of management action is critical because there is general agreement for the existence of a strong link between resilience and sustainability [10]. Successfully increasing the resilience of natural systems may therefore have important implications for human welfare in the face of global climate change.

In this Perspective, we will argue that the expectation of increased resilience of natural communities to climate change through the reduction of local stressors may be fundamentally incorrect, and that resilience-focused management may, in fact, result in greater vulnerability to climate impacts. We illustrate our argument using coral reefs as a model. Coral reefs are in an ecological crisis due to climate change and the ever-increasing magnitude of human impacts on these biodiverse habitats [11],[12]. These impacts stem from a multiplicity of local stressors, such as fishing, eutrophication, and sedimentation. It is therefore not surprising that the concept of resilience—to climate change in particular—is perhaps more strongly advocated as an underpinning of management for coral reefs than for any other ecosystem [9],. Marine reserves or no-take areas, the most popular form of spatial management for coral reef conservation, are widely thought to have the potential to increase coral reef resilience [11],[13],[14],[17]. But do they really?

## The Conventional View of Resilience

**Figure 1:**
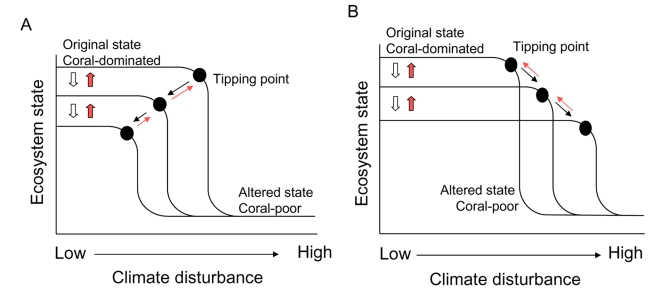
Managing coral reefs for resilience to climate change. A. The conventional view of resilience. Natural communities are highly resilient to climate change, i.e., the tipping point (black circle) leading to an alternative ecosystem state is far to the right and attained only at high levels of climate disturbance. As chronic anthropogenic disturbances gradually degrade the original ecosystem (open block arrows), the tipping point in response to climate change gradually shifts to the left (black arrows), making the ecosystem less resilient to climate disturbance. Management that seeks to control local anthropogenic disturbances should reverse degradation (red block arrows), shifting the tipping point back to the right, towards higher resilience (red arrows). B. A possible counter-intuitive effect of managing coral reefs for resilience to climate change. If the effect of chronic anthropogenic disturbances, which gradually degrade the original ecosystem (open block arrows), is to remove disturbance-sensitive individuals and/or species, the tipping point in response to climate change will gradually shift to the right (black arrows), making the ecosystem more resilient to climate disturbance. Management that seeks to control local anthropogenic disturbances and reverse degradation (red block arrows) will inadvertently shift the tipping point back to the left, towards lower resilience (red arrows) to climate disturbance.

The concept of managing for resilience is underpinned by the notion that unstressed coral communities are highly resilient to climate change and that human-induced degradation erodes the ability of coral reefs to resist the impacts of climate disturbance, tipping degraded reefs into alternative, less desirable states sooner than pristine ones [13]. This conventional view is illustrated in the simple conceptual model shown in Figure 1, which depicts the potential relationships between ecosystem state and the strength of climate disturbance. Here, we focus on corals—the three-dimensional reef builders that are the foundation species for most reef communities [18]—thus ecosystem state could be measured as coral cover or coral species diversity, whereas climate disturbance can incorporate both a change in mean temperature or increased variability [19].

The model implies that more pristine coral communities will cross a tipping point and subsequently shift into an alternative ecosystem state—usually dominated by fleshy macroalgae [13] but other alternative states are possible [20]—only at high levels of climate disturbance (Figure 1A). As non-climatic, local disturbances degrade the original ecosystem (Figure 1A; open block arrows), the tipping point in response to climate change shifts to the left (Figure 1A; black arrows), making the ecosystem less resistant to climate disturbance. Management that seeks to control local stressors and reverse degradation (Figure 1A; red block arrows) is therefore expected to increase resilience by shifting the tipping point back to the right and keeping reefs further away from this ecological precipice (Figure 1A; red arrows).

If resilience to climate change varies in relation to ecosystem state as depicted in Figure 1A, then two general predictions arise. First, coral communities exposed to local or chronic disturbance should be more susceptible to climate change than less degraded communities. Second, corals in areas with management to control local disturbances should be less susceptible to climate perturbations than those in areas without similar management. We evaluate briefly the empirical evidence for each prediction below.

### 

*Are degraded communities more susceptible to climate change impacts?*

Ecologists are increasingly aware that, in a variety of ecosystems, species loss following disturbance is non-random [3],[21],[22]. On coral reefs, selective mortality following disturbance has a direct impact of coral community structure, by changing the absolute and relative abundances of coral species [23]. Shifts in community assemblages have been observed in the aftermath of diverse natural and anthropogenic disturbances, including storms [23]–[25], pollution [26], sedimentation [27]–[31], fishing [32], disease [27], and coral predator outbreaks (e.g., crown-of-thorns sea stars, [33]).

The general trend of such community shifts is the loss of coral species with stress-sensitive life histories and increases in dominance (both in terms of absolute and relative abundance) of stress-tolerant species that survive the disturbance and of opportunistic species that rapidly colonize following a disturbance. In the Indo-Pacific region, this trend is exemplified by the replacement of stress-sensitive branching and plating coral genera, such as *Acropora* and *Montipora*, by stress-tolerant massive corals such as massive *Porites*, and the faviids *Platygyra* and *Favia* [26],[28],[34]. In the Caribbean, the primary reef-building corals, *Acropora* and *Montastrea* species, have been replaced by “weedy” coral species that form small colonies, grow quickly, and are short-lived [35],[36]. For example, the relative abundance of “weedy” *Porites astreoides* has increased significantly over the past four decades [37] as coral cover—an acknowledged sign of reef degradation—has declined across the region [38]. Disturbed Caribbean reefs have also been shown to converge to communities dominated by *Agaricia*, whose opportunistic life-history and high environmental tolerance have been suggested to explain its persistence in degraded reef habitats [27].

The conventional view of resilience predicts that these shifted or “degraded” coral assemblages should be more vulnerable to climate change. The fact that thermally induced coral bleaching events—currently the most visible manifestation of climate change on coral reefs—are increasing in frequency and extent [11],[39] on reefs that are globally degraded [38],[40] could be taken as supporting evidence. However, this signal is confounded by increasing sea surface temperature anomalies over time [11],[19]. To our knowledge, there is no evidence to suggest that bleaching events are now triggered by lower temperatures than they were in the past, when coral reefs were generally less degraded (Perry et al., unpublished data). Nearly “pristine” reefs can experience high bleaching-induced mortality (e.g., Phoenix Islands, [41]). In fact, isolated reefs, such as those of Palmyra in the Line Islands, can bleach as severely as more impacted reefs (e.g., in American Samoa, Fiji, and the Philippines), despite the fact that they experience temperature regimes that are not hotter (or cooler) [42]. Furthermore, the apparently higher bleaching resistance of one coral species (*Montastrea faveolata*) from an isolated Belizean atoll with low anthropogenic stress can also be ascribed to milder heat stress on these reefs than on more degraded reefs [43].

### 

*Are protected communities less susceptible to climate change?*

Marine reserves (aka no-take areas) are the most popular tool for controlling local stressors, primarily fishing, on coral reefs [9],[17],[18]. They are known to have positive effects on the abundance and diversity of a variety of taxa within their boundaries [44]. High species diversity within marine reserves is expected to provide protected reefs with ecological insurance and increased functional redundancy, which is commonly assumed to increase resilience to disturbance events [15],[45]. Yet, marine reserves do not reduce the frequency or intensity of thermally induced coral bleaching [9],[14],[46] or bleaching-induced coral mortality compared to unprotected areas [47]–[49]. In fact, thermal stress can cause proportionally greater coral mortality of protected than unprotected corals [19],[47]–[49]. This effect is probably due to the different coral species composition between protected and unprotected sites. Indeed, the higher abundance of thermally sensitive corals, such as *Acropora* and *Montipora*, within marine reserves is associated with the increased susceptibility of protected coral assemblages to climate disturbances [19],[47],[48]. Such differences in coral assemblages are not likely to be due to site selection bias [47],[50], but to the effects of protection. There is also no evidence that marine reserves are currently located in areas that are less likely to get hot [51]. Finally, there is no expectation that marine reserves will alleviate the impacts of ocean acidification on corals [9].

The lack of observable effects of protection on the ability of corals to resist thermal disturbance could be explained if marine reserves are failing to return degraded coral reefs to less degraded states (i.e., not actually moving up the Y axis in Figure 1). While this may sometimes be the case [46],[52], many reserves show higher coral recruitment [53] or coral species diversity [32], maintain coral cover [32],[50], and increase rates of coral recovery [54], with concomitant declines in macroalgal cover [53],[54]. Thus, marine reserves benefit corals, but the dominant impact of climate change can override any advantage provided by protection from fishing [47].

## Resilience in a Disturbed World: An Alternative View

The two predictions of the conventional view of ecological resilience are poorly supported by empirical evidence pertaining to coral reefs. We believe that the selective culling of disturbance-sensitive taxa by local stressors can explain why more intact reef communities do not appear to be more resilient to climate disturbance. If a species' tolerance to a non-climatic disturbance is correlated with its tolerance to climatic impacts (e.g., positive co-tolerance, [55]), then degradation can actually increase the abundance of disturbance-tolerant species within a community [26],[28] and thus the ability of an ecosystem to resist the impacts of climate disturbance.

This alternative view, which is more consistent with the majority of empirical observations, is depicted in Figure 1. Thus, with continued degradation caused by local stressors, altered communities become composed of disturbance-tolerant species and the tipping point in response to climate change will shift to the right (Figure 1B; black arrows), making the ecosystem *more* resilient to climate disturbance. Management that seeks to control local anthropogenic disturbances and reverse degradation (Figure 1B; red block arrows) will inadvertently shift the tipping point back to the left, towards lower resilience (Figure 1B; red arrows) to climate disturbance. Thus, management that controls local stressors to reverse degradation and recover original species assemblages will actually increase the proportion of sensitive taxa within the assemblage, and may effectively *decrease* ecosystem resilience to climate change.

Note that the alternative states depicted in Figure 1 are not assumed to be stable. Moreover, our conceptual model works with or without thresholds. If ecosystem state declines linearly with climate disturbance, we expect that the slope of this relationship will decrease as degradation increases (i.e., as the intercept decreases).

## Resistance versus Recovery and the Role of Protected Areas in a Changing Climate

It is widely held that reducing local stressors will mitigate the impacts of global stressors, such as climate change. We have suggested here that this assumption may be fundamentally flawed, at least in terms of one facet of resilience, namely the ability of communities to resist climate-induced stress. The other facet of resilience is recovery. There is growing evidence that protected or less degraded reefs return more quickly to their original state following a range of disturbances (including thermal stress) than unprotected or more degraded reefs (e.g., [43],[54]; but see [32],[47]). Thus, the alleviation of local stressors can potentially enhance reef recovery from climate change impacts.

Conservationists may therefore have to choose between bolstering ecosystem resistance and ecosystem recovery because management action, such as the implementation of protection, should be expected to promote the latter but hinder the former. We would argue that the focus should be on resistance rather than recovery for two reasons. First, the frequency of extreme climatic events is expected to increase under most climate change scenarios [11],[56], thus the window available between climate disturbances may be less than the time needed for reefs to recover. Second, not all climate disturbances will be acute. In response to chronic climate stressors, such as globally increasing sea surface temperatures and ocean acidification [11], there will be no role for recovery in reef resilience. Enhancing reef resistance to climatic stress is therefore a better long-term goal.

Can coral reefs, or any other ecosystem, actually be managed for resistance to climate change? Our conceptual model implies that ecosystem resistance (i.e., or the extent to which the tipping point is shifted to the right; Figure 1B) should co-vary with increasing degradation. This is true only up to a point. Beyond a threshold level of degradation, changes in species composition and interactions may become irreversible, impairing ecosystem function and (both aspects of) resilience. Near-shore communities of the Great Barrier Reef may be an example. These reefs have been exposed to heavy disturbances from sedimentation, nutrient pollution, and cyclones, and may be at that point where their ability to resist coral bleaching has been surpassed [57]. Here, reefs with a high probability of experiencing heavy nitrogen-rich terrestrial runoff appear to bleach at lower threshold temperatures than reefs in more permanently oligotrophic oceanic locations [58], leading to the suggestion that management to improve water quality could increase bleaching resistance [59]. On severely degraded reefs such as these, managing for resistance may be unsuccessful and removing local stressors could offer the only hope for recovery in between disturbances. The challenge for managers will be to identify the levels of local stress that maximize ecosystem resistance.

Cynics may view our argument as a justification for advocating against marine protected areas, but this would be short-sighted. While protected areas may not increase ecosystem resistance to climate change, these areas can help to accelerate recovery and effectively act as an insurance policy for biodiversity, by preserving sensitive and specialized species that cannot persist in disturbed and altered environments. However, to fulfill their insurance role, protected areas will need to be placed in locations that are predicted to escape the brunt of climate change [9],[16],[51]. Without a strategically distributed network of protected areas, communities of the future will likely be limited to weedy and disturbance-tolerant generalist species that may or may not preserve ecosystem function and services. Moreover, these altered assemblages may only provide resilience up to a point, as even thermally tolerant species will have stress limits that may be exceeded by ongoing ocean warming and acidification [11].

Climate change is likely to be the dominant driver of ecological change in the 21st century and removing local stressors may not be enough to maintain biological diversity. We believe that there is hope for the survival of natural ecosystems in a changing climate. However, the emphasis of the global conservation agenda needs to shift substantially from dealing with tractable, local stressors to tackling the more fundamental problem of curbing atmospheric CO_2_ emissions.
